# Incorporating Pooled Donkey Milk from Autochthonous Balkan and Banat Donkey Breeds into Traditional Dairy Products: Effects on Technological Properties, Nutritional Profile, and Sensory Acceptability of *Podliveni* Cheese

**DOI:** 10.3390/ani16101449

**Published:** 2026-05-09

**Authors:** Dragana Ljubojević Pelić, Suzana Vidaković Knežević, Nenad Popov, Slobodan Knežević, Jelena Vranešević, Miloš Pelić, Milica Živkov Baloš

**Affiliations:** 1 Scientific Veterinary Institute “Novi Sad”, 21000 Novi Sad, Serbia; 2 Institute of Food Technology, University of Novi Sad, 21000 Novi Sad, Serbia

**Keywords:** autochthonous donkey breeds, donkey milk, traditional cheese production, value-added products

## Abstract

*Podliveni* cheese is a traditional fresh cheese produced in Serbia, typically made from fresh cow’s milk. Donkey milk is recognized for its nutritional benefits, particularly its hypoallergenic properties; however, its use in cheese production is partially limited due to its specific protein composition and low casein content. In addition, information in the scientific literature regarding its application in cheese production remains limited. In this study, *Podliveni* cheese was produced from raw cow’s milk, while in a second experimental group, 30% milk from autochthonous Balkan and Banat donkey breeds was added to obtain a value-added *Podliveni* cheese. The selected proportion (30%) was based on previous studies using lower inclusion levels (10% and 20%), which demonstrated measurable but limited effects on cheese properties. The technological production process was identical in both groups and is described for each type of cheese. Microbiological parameters analyzed included total lactic acid bacteria (LAB), *Enterobacteriaceae*, *Escherichia coli*, coagulase-positive staphylococci (CPS), *Salmonella* spp., and *Listeria monocytogenes*. Sensory analysis was conducted using a five-point hedonic scale with a panel of 21 participants (male and female, aged 20–60 years). The following chemical composition parameters were also evaluated: dry matter, fat content, fat in dry matter, fat-free dry matter, protein, ash, pH, and salt. The content of essential minerals and trace elements was determined, including Ca, P, Na, K, Mg, Zn, Cu, Fe, and the Ca/P ratio. The addition of donkey milk significantly affected curd formation, which required six times longer compared to cheese produced exclusively from raw cow’s milk. Furthermore, the inclusion of donkey milk reduced cheese yield and resulted in increased whey separation during storage, indicating reduced water-holding capacity. No statistically significant differences were observed in microbiological parameters, and pathogenic bacteria (*Salmonella* spp. and *Listeria monocytogenes*) were not detected in either cheese. No significant differences were observed in most sensory attributes, except for texture. Conversely, the inclusion of donkey milk significantly affected the majority of chemical parameters and the mineral composition of the cheese. The addition of donkey milk resulted in a significant decrease (*p* < 0.05) in fat, fat in dry matter, fat-free dry matter, Ca, P, K, Zn, Cu content and the Ca/P ratio, while a significant increase (*p* < 0.05) was observed in dry matter, protein, salt, Na, Mg, and Fe content. The incorporation of donkey milk represents an innovative approach that expands the range of traditional cheeses without compromising the absence of tested pathogenic bacteria and preserving traditional production practices, simultaneously offering new value-added products. Further research is required to better understand the health benefits associated with the inclusion of donkey milk in cheese production. This study contributes to expanding knowledge on the use of donkey milk and supports the conservation of autochthonous breeds and the improvement of human health.

## Simple Summary

*Podliveni* cheese is a traditional fresh cheese produced in Serbia, typically made from cow’s milk. Donkey milk is valued for its nutritional quality and hypoallergenic properties, but its use in cheese production is limited. This study evaluated the effect of adding donkey milk to *Podliveni* cheese on production characteristics, microbiological safety, and sensory acceptance. Cheese was produced from raw cow’s milk and from a mixture containing cow’s and 30% donkey milk obtained from autochthonous Balkan and Banat donkey breeds. The same technological process was applied in both cases. Microbiological safety, chemical composition, mineral content, and sensory properties were assessed. The addition of donkey milk significantly increased curd formation time and reduced cheese yield, while also affecting cheese texture. However, no differences were observed in microbiological safety or overall sensory acceptance. The addition of donkey milk resulted in a lower fat content and higher protein levels, as well as changes in the mineral composition of the cheese. These findings indicate that donkey milk can be successfully incorporated into traditional cheese production, creating microbiologically safe, value-added dairy products. This approach supports the preservation of traditional foods, promotes the use of autochthonous donkey breeds, and may contribute to improved nutritional quality in dairy products.

## 1. Introduction

*Podliveni* cheese is a traditional homemade type of soft, fresh cheese frequently produced in rural households in Serbia. It is obtained through a simple artisanal production process using raw fresh cow’s milk, rennet, and salt. During the production process, milk is coagulated with rennet, after which the curd is drained through gauze or cloth to remove whey, and finally salted. The cheese is characterized by a mild flavor and soft texture. It can be produced from whole or partially skimmed milk, either raw or pasteurized, sometimes with the addition of herbs such as peppers and parsley leaves [1]. Due to its traditional production method and strong geographical identity, *Podliveni* cheese can be considered a product with characteristics of a geographically recognizable [1]. Traditional cheeses represent an important part of cultural heritage and regional identity [2,3], while also offering opportunities for technological innovation and product diversification.

In recent years, consumer demand has increasingly shifted toward high-quality traditional foods with added nutritional value. Consumers are increasingly seeking new, innovative and healthier fresh dairy products that combine traditional production methods with improved nutritional profiles [4]. The development of functional dairy products based on traditional technologies represents an important strategy for improving both nutritional quality and sensory acceptance. One promising approach to meeting this demand is the incorporation of milk from alternative dairy species into conventional dairy products in order to enhance their nutritional value and diversify the dairy market.

The nutritional benefits of donkey milk have been recognized since ancient times [5]. What distinguishes it from the milk of other domestic animals is its similarity to human milk, primarily due to its specific chemical composition, particularly in terms of total protein content, amino acid profile and lactose content [6,7,8]. Owing to the increasing prevalence of allergies to cow milk proteins, donkey milk has found its place as an alternative in the nutrition of newborns as well as other sensitive population groups [9,10]. The characteristic composition of donkey milk, including its low content of milk fat and proteins, distinctive mineral composition, and the presence of bioactive components such as lysozyme, lactoferrin, lactoperoxidase, and immunoglobulins [11,12], may contribute to increasing the nutritional value of products made from donkey milk. Furthermore, these components contribute to its antimicrobial and immunomodulatory properties [8,13] and may positively influence the microbiological and sensory characteristics of dairy products produced from donkey milk [14,15]. Due to these properties, donkey milk is increasingly considered as a valuable functional ingredient for the development of innovative dairy products.

However, despite these recognized nutritional benefits, the specific composition of donkey milk also leads to technological challenges during cheese production [15]. In particular, coagulation is more difficult due to the significantly lower total casein content in donkey milk, which is about 7.8 g/kg, compared to approximately 26 g/kg in cow milk [11]. This difference significantly affects curd formation and cheese yield. Various technological processing strategies can be applied to overcome this issue, and several studies have already reported the successful production of cheese from donkey milk using different technological approaches [14,16,17,18,19,20,21,22]. Such approaches include ultrafiltration to increase protein concentration, the use of enzymes such as transglutaminase to enhance protein cross-linking, optimization of coagulation conditions, and the selection of appropriate coagulants such as calf rennet. However, scientific information on the application of donkey milk in traditional fresh cheese varieties remains limited, particularly regarding its incorporation into region-specific artisanal cheeses. In the present study, no technological modifications were applied intentionally, as the aim was to evaluate the feasibility of incorporating donkey milk into traditional *Podliveni* cheese while preserving the original artisanal production method.

The Balkan donkey, an autochthonous breed in Serbia, has been the subject of scientific research in recent years [14,23,24,25]. More recently, studies have also been conducted on the Banat donkey [16,26,27], which, together with the Balkan donkey, is bred in the Special Nature Reserve Zasavica. Both breeds represent valuable genetic resources and contribute to the preservation of biodiversity and sustainable livestock production.

In our previous research, we investigated the effect of adding milk from these two donkey breeds during the production of *pasta filata*-type rolled cheese, where donkey milk was added at levels of 10% and 20% relative to cow milk [16]. The results demonstrated that even these inclusion levels significantly (*p* < 0.05) affected the chemical composition and mineral profile of the cheeses, resulting in decreased fat, fat in dry matter, calcium content, and calcium-to-phosphorus ratio, alongside increased ash, salt, sodium, and potassium contents. Furthermore, sensory evaluation confirmed that cheeses containing donkey milk were clearly distinguishable from those produced exclusively from cow milk, including a strong potential for the development of value-added dairy products. These findings suggested that increasing the proportion of donkey milk may further intensify its influence on cheese characteristics. Accordingly, the present study employs a higher inclusion level (30%) to provide a more comprehensive evaluation of its impact on the physicochemical, nutritional, and sensory properties, while assessing its suitability for cheese production. Building upon these findings, the present study investigates the possibility of incorporating donkey milk into another traditional Serbian cheese type, *Podliveni* cheese. The selection of a 30% inclusion level was based on the need to evaluate the upper practical limit of donkey milk addition that still allows successful cheese production, while simultaneously assessing the potential for enhanced nutritional value associated with a higher proportion of donkey milk.

To the best of our knowledge, this study represents the first attempt to incorporate donkey milk from the autochthonous Balkan and Banat donkey breeds into the production of traditional *Podliveni* cheese. The aim of this study was therefore to evaluate the technological feasibility of producing *Podliveni* cheese with the addition of 30% donkey milk and to comprehensively assess its microbiological safety, physicochemical composition, mineral profile, and sensory characteristics. This research contributes to the development of value-added traditional dairy products while simultaneously supporting the preservation of indigenous donkey breeds, promoting biodiversity, and encouraging sustainable rural development and food system sustainability.

## 2. Materials and Methods

### 2.1. Milk Collection

Raw donkey milk was sampled according to the procedure described in our two previous studies [16,27]. Briefly, donkey milk was collected in December 2024 during morning milking from animals kept in the Special Nature Reserve Zasavica, located in northern Serbia. The milk represented pooled samples collected from Balkan and Banat donkey breeds reared under identical feeding, management, and environmental conditions under extensive farming conditions. Since the two breeds are kept together in the reserve, and currently there is no technical possibility to collect milk separately from each breed; therefore, the obtained milk represented a pooled sample from both breeds. Since both breeds are maintained under identical feeding, management, and environmental conditions, pooled sampling was considered appropriate to obtain representative milk samples under practical production conditions. A detailed description of the animals, lactation management, and production conditions has been previously reported [27]. Cow’s milk was obtained from a small-scale Holstein–Friesian dairy farm in northern Serbia. All collected milk samples were stored at 4 °C prior to cheese production. Physicochemical characteristics of the raw milk used for cheese production were previously determined and reported in our earlier study [16], as the same milk batch was used for both investigations. In addition, total mesophilic bacteria counts were determined in the present study to assess the microbiological quality of the raw milk. These data are summarized in Table 1.

### 2.2. Process of Podliveni Cheese Production

Two groups of *Podliveni* cheese, a traditional fresh cheese produced in Serbia, were manufactured in a small-scale dairy plant located in northern Serbia. One group was produced from raw cow’s milk, while the other was made from a mixture of raw cow’s and raw donkey’s milk. The technological process was identical for both groups, except for coagulation time, which differed due to the presence of donkey milk. A total of 10 L of milk was used for cheese production, consisting of 7 L of cow’s milk and 3 L of donkey’s milk. No prior milk standardization was performed. The milk was heated to 40 °C, after which 0.3 g of rennet (Caglificio Clerici, Como, Italy; with a declared activity of 1170 IMCU/g) was added and thoroughly mixed. The milk was then left undisturbed until coagulation occurred. The coagulation time for cow’s milk was 30 min, whereas the coagulation time for the mixture of cow’s and donkey’s milk was approximately six times longer under the same conditions. After coagulation, the curd was cut into cubes of approximately 2 cm^3^ using a stainless-steel curd knife. Cutting was performed uniformly in vertical and horizontal directions under identical conditions for all batches to ensure consistency in curd size and whey separation. The curd was then further gently stirred until particles reached approximately pea size, followed by partial whey removal by decanting, and subsequently left to stand for an additional 30 min to facilitate further whey separation. The obtained curd was transferred into a mold lined with cotton cheesecloth and left to drain for 12 h at room temperature. The cheese was then cut into pieces and lightly dry-salted by hand using table salt. Salting was performed by manual dry application of NaCl. To ensure consistency and minimize variability among samples, salting was carried out by the same operator under identical conditions immediately after curd drainage and portioning. The cheese was placed in plastic containers and stored at 4 °C until analysis, which was performed the following day.

### 2.3. Microbiological Analysis

A total of 25 g of each cheese sample was homogenized in 90 mL of sterile peptone water (Biokar Diagnostics, Beauvais, France) using a Stomacher device (Mayo International SRL, Novate Milanese, Italy) for 2 min. Subsequently, serial decimal dilutions were prepared, followed by microbiological analyses. Total lactic acid bacteria (LAB) were enumerated on de Man, Rogosa, Sharpe agar (HiMedia Laboratories Pvt. Ltd., Mumbai, India) after 72 h incubation at 30 °C [28]. *Enterobacteriaceae* were enumerated on violet red bile glucose agar (VRBGA, CM1082, Oxoid, UK), incubated at 37 °C for 24 h [29]; β-Glucuronidase-positive *Escherichia coli* were quantified on tryptone bile glucuronide agar (TBX, CM0945, Oxoid, UK), following incubation at 44 °C for 24 h [30]. Coagulase-positive staphylococci (CPS) were assessed on Baird Parker agar (Biokar Diagnostics, Beauvais, France) after incubation at 37 °C for 24–48 h [31]. Detection of *Salmonella* spp. was performed in accordance with ISO 6579-1 [32], while the detection of *Listeria monocytogenes* was carried out following ISO 11290-1 [33].

### 2.4. Chemical Analysis

#### 2.4.1. Physicochemical Analysis

The dry matter content was determined by oven drying the samples at 102 °C in a laboratory drying oven, following the ISO standard method [34]. Fat content was assessed using the acid-butyrometric method [35], while fat in dry matter was calculated from the measured moisture and fat content. Total nitrogen was quantified using the Dumas combustion method, performed on a Rapid N Exceed Analyzer (Elementar, Langenselbold, Germany), and the protein content was calculated using a nitrogen-to-protein conversion factor of 6.38 [36]. This conversion factor is recommended by ISO standards for milk and milk products and is commonly applied in studies evaluating protein content in donkey milk and dairy products containing donkey milk, due to the absence of a universally established donkey-specific conversion factor.

The pH value was measured at 20 ± 0.5 °C with a calibrated electronic pH meter (Consort C 830, Turnhout, Belgium), using standard buffer solutions. Ash content was determined via dry ashing in a muffle furnace at 550 °C, according to AOAC guidelines [37].

#### 2.4.2. Essential Minerals and Trace Elements Analysis

1. Sample Preparation

Cheese samples were processed for ICP-MS analysis using an acid digestion procedure performed in a Microwave Labstation Ethos system (Milestone s.r.l., Sorisole, Italy). To ensure representativeness, cheese samples were thoroughly homogenized prior to digestion. Approximately 10 g of each sample was homogenized using a laboratory blender until a uniform mass was obtained. The homogenized material was then subjected to a quartering procedure to obtain a representative subsample, from which 1 g was accurately weighed for microwave digestion and subsequent mineral analysis. Specifically, 1 g of each cheese sample was combined with 8 mL of diluted nitric acid (prepared in a 2:1 ratio of 65% *w*/*w* HNO_3_ to deionized water) and 2 mL of hydrogen peroxide (30% *w*/*w*). The mixture underwent microwave-assisted digestion at 180 °C under 40 bar pressure for 30 min. After digestion, the resulting clear solution was transferred quantitatively into a volumetric flask and brought to a final volume of 25 mL with deionized water. A reagent blank was prepared using the same procedure, excluding the sample. All preparations were carried out in triplicate.

2. Equipment and Mineral Element Analysis

The elemental analysis was performed using an Agilent 7700 ICP-MS instrument (Agilent Technologies, Santa Clara, CA, USA), equipped with an Octopole Reaction System (ORS) to reduce spectral interferences. Internal standards were prepared using single-element solutions of Ge, Rh, Lu, and Ir, obtained from CPI International (Amsterdam, The Netherlands). Calibration standards for Ca, P, Na, K, Mg, Fe, Zn, and Cu were prepared daily from single-element stock solutions (AccuTrace™ Reference Standards, New Haven, CT, USA). Sodium chloride (salt) was calculated as sodium content × 2.5 (%).

### 2.5. Sensory Evaluation

Sensory evaluation of the cheeses was conducted at the Scientific Institute of Veterinary Medicine “Novi Sad” by 21 employees, including both male and female participants aged between 20 and 60 years. Prior to the evaluation, each participant completed a questionnaire collecting information on gender, age, and previous experience with donkey milk and/or cheeses made from donkey milk [16]. In addition, the panelists were familiarized with the evaluation procedure, the sensory scale, and the assessed attributes, which included color, texture, aroma, taste, and overall liking. For the sensory analysis, the cheeses were coded with random three-digit numbers and served in 30 g portions. The samples were evaluated using a five-point hedonic scale ranging from 1 = dislike extremely to 5 = like extremely.

### 2.6. Statistical Analysis

Each experimental group consisted of one cheese sample, and all analytical measurements were performed in triplicate (technical replicates). The reported results represent the mean ± standard deviation of replicate measurements. To assess differences between groups, a one-way ANOVA using the F-test was employed. The statistical analysis was conducted with Microsoft Excel 2007. Differences were deemed statistically significant at a probability level of less than 0.05 (*p* < 0.05). The critical F-value for this analysis was calculated based on the degrees of freedom corresponding to between-group variation (df_1_ = 1) and within-group variation (df_2_ = 4) at the 0.05 significance level. According to the F-distribution table, this critical value is approximately 7.71. Therefore, if the observed F-statistic is greater than this threshold, the differences between groups are considered significant.

## 3. Results and Discussion

### 3.1. Podliveni Cheese Production

During the production of *Podliveni* cheeses, the incorporation of donkey milk significantly affected the coagulation process. The cheese produced from a mixture of cow’s and donkey’s milk required approximately six times longer for curd formation compared to cheese produced exclusively from cow’s milk. The prolonged coagulation time can be explained by the specific protein composition of donkey milk. It is well known that donkey milk contains significantly lower levels of casein compared to cow’s milk [11], which directly affects the coagulation process and curd formation [38,39]. The reduced casein content contributes to weaker gel formation and slower curd development [15]. The coagulation time of cheese produced exclusively from cow’s milk was 30 min, whereas the coagulation time of *Podliveni* cheese produced from a mixture of cow’s and donkey’s milk was 2 h.

In addition, the inclusion of donkey milk resulted in a lower cheese yield. This observation is consistent with previous studies reporting that lower dry matter, casein, fat and protein content of donkey milk compared to cow’s milk negatively affects cheese yield [17,18,40]. In the present study, the yield of cheese produced exclusively from cow’s milk was 1.37 ± 0.05 kg, whereas the yield of *Podliveni* cheese produced from a mixture of cow’s and donkey’s milk was 1.10 ± 0.09 kg, showing a statically significant difference (*p* < 0.05).

**Figure 1:**
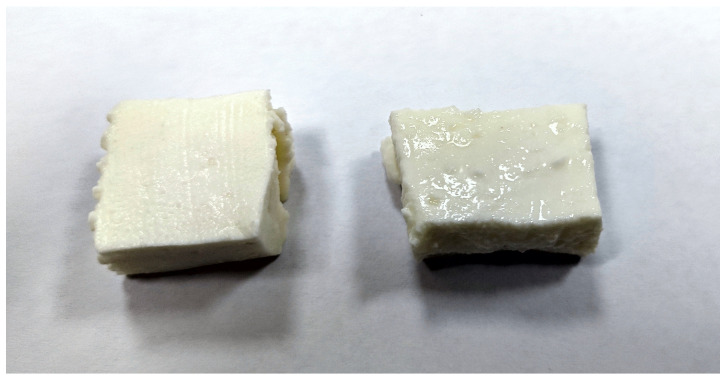
*Podliveni* cheese piece made from raw cow’s milk (**left**) and cheese piece made from 70% raw cow’s milk and 30% raw donkey’s milk.

Furthermore, during storage, cheese produced with the addition of donkey milk released a greater amount of whey and exhibited a visibly moister appearance (Figure 1). The increased whey separation during storage may be associated with the formation of a weaker protein network and reduced water-holding capacity of the curd [41].

Moreover, the texture of the cheese was softer and more delicate, both to the touch and during slicing. Similar textural changes have been reported in previous studies in which donkey milk was incorporated into cheese production [17,18,19,40].

### 3.2. Microbiological Status of Podliveni Cheese

The microbiological status of *Podliveni* cheese is presented in Table 2. No statistically significant differences were observed between cheeses for any of the analyzed microbiological parameters (*p* > 0.05). However, *Enterobacteriaceae* counts showed a tendency toward significance (*p* = 0.059).

Various microorganisms may enter milk during milking from different environmental sources, including soil, air, animal feed, worker’s hands, milking equipment, and teat surface [42,43]. For this reason, maintaining appropriate hygiene of animals and their environment, proper sanitation of milking equipment, and adherence to hygienic practices during milking and milk storage are of critical importance [44]. Milk from healthy animals is considered sterile within the udder. However, after milking it becomes an excellent medium for microbial growth [45,46]. Because pathogenic microorganisms may occur, cheeses produced from raw milk are generally considered high-risk foods from a food safety perspective [47,48].

*Podliveni* cheese has a relatively high moisture content, low salt concentration, and a pH value favorable for microbial growth. In addition, during its production the milk is heated to approximately 40 °C prior to the addition of rennet, which may create favorable conditions for the development of microorganisms. The commercial rennet used in cheese production contributes to acidification and a reduction in pH to levels that slow or inhibit the growth of pathogenic microorganisms [16].

In the present study, the pathogenic microorganisms *Salmonella* spp. and *L. monocytogenes* were not detected in any of the cheese samples. This finding indicates that appropriate hygienic conditions were maintained during cheese production and may also suggest a potential contribution of the antimicrobial properties of donkey milk [25,49], which was incorporated at a level of 30% in this study. Similar findings have been reported in previous studies. In our previous research on *pasta filata* rolled cheese produced with the addition of 10% and 20% donkey milk, these pathogens were also not detected [16]. Šarić et al. [14] also reported that *Salmonella* spp. and *L. monocytogenes* were not detected in extra-hard cheese produced from a mixture of donkey and caprine milk. Likewise, Faccia et al. [18] reported the absence of these pathogens in cheese produced from a mixture of donkey and goat milk. The absence of *Salmonella* spp. and *L. monocytogenes* confirms the microbiological safety of the cheeses despite the use of raw milk.

The number of LAB in *Podliveni* cheese produced solely from cow’s milk was 8.83 ± 0.32 log_10_ CFU/g, while in cheese produced with the addition of 30% donkey milk it was slightly higher, reaching 8.93 ± 0.05 log_10_ CFU/g. However, the difference was not statistically significant. In our previous study evaluating total mesophilic bacterial counts in rolled cheese, the addition of donkey milk also did not significantly affect this parameter [16]. In that study, total mesophilic counts ranged from 5.55 to 5.58 log_10_ CFU/g, which is considerably lower than the values of LAB observed in the present study. This difference is most likely related to technological differences, particularly the fact that during rolled cheese production temperatures of approximately 80 °C are reached, whereas such high temperatures are not applied during *Podliveni* cheese production. In addition, the presence of indigenous LAB in raw milk should not be overlooked. Even when present in low numbers, these microorganisms can proliferate under favorable conditions of temperature and nutrient availability, leading to high LAB counts in the formed curd and the final cheese [50]. To the best of our knowledge, there are no available data on LAB counts in similar types of cheese produced from raw donkey milk. In the study conducted by Šarić et al. [14], LAB counts were 4.80 ± 0.1 log_10_ CFU/g. However, in that study, the milk was pasteurized and the cheese underwent a ripening process lasting six months. It is well known that LAB can inhibit the growth of pathogenic and spoilage microorganisms by increasing acidification [51] and producing antimicrobial compounds [52]. Considering this, the relatively high LAB counts observed in the present study may contribute to the microbiological stability and quality of the cheese [53].

*Enterobacteriaceae* are important, not only as indicators of hygiene during milking and cheese production, but also because some members of this family are potential pathogens capable of causing foodborne illness [54]. In addition, they may negatively affect cheese quality due to their ability to metabolize proteins and produce gas [55].

*Enterobacteriaceae* counts in cheese produced solely from cow’s milk were 4.59 ± 0.25 log_10_ CFU/g, while slightly higher values were recorded in cheese produced with the addition of 30% donkey milk, 4.94 ± 0.32 log_10_ CFU/g, with a tendency toward statistical significance (*p* = 0.059). In our previous study on rolled cheese produced with donkey milk, *Enterobacteriaceae* counts ranged from 2.37 to 2.67 log_10_ CFU/g and no statistically significant effect of donkey milk addition was observed [16]. The lower values reported in rolled cheese compared to those obtained in the present study for *Podliveni* cheese may be explained by the higher temperatures reached during rolled cheese production (approximately 80 °C), whereas *Podliveni* cheese production involves heating milk only to about 40 °C. The results obtained in our study are within the reported range for *Enterobacteriaceae* in cheeses produced from raw milk. In studies on cheeses produced from raw milk in Switzerland, *Enterobacteriaceae* counts in 13.7% of the cheeses ranged from 2 to 4.94 log_10_ CFU/g [56]. In another study [57], *Enterobacteriaceae* counts reached values between 5 and 8 log_10_ CFU/g. Furthermore, in a study conducted by Sakaridis et al. [58], the mean *Enterobacteriaceae* count in raw curd was 6.92 log_10_ CFU/g.

*E. coli*, a member of the *Enterobacteriaceae* family, is commonly used as an indicator of fecal contamination [54,59] and may also be associated with foodborne illness following the consumption of raw milk cheeses [60].

In the present study, the number of *E. coli* in *Podliveni* cheese produced exclusively from cow’s milk was 3.03 ± 0.23 log_10_ CFU/g, while slightly lower values were observed in cheese produced with the addition of 30% donkey milk (2.98 ± 0.124 log_10_ CFU/g), although the difference was not statistically significant. Faccia et al. [18] reported *E. coli* levels below 1 log_10_ CFU/g in fresh cheese produced with the addition of donkey milk. In our previous study on rolled cheese, *E. coli* counts were 1.89 log_10_ CFU/g in cheese produced solely from cow’s milk, while the addition of 10% and 20% donkey milk increased this value to 2.09 and 2.29 log_10_ CFU/g, respectively; however, no statistically significant differences were observed in that study either [16]. The lower values recorded in rolled cheese compared to *Podliveni* cheese are likely associated with the higher temperatures reached during rolled cheese production. Our results are consistent with previously reported values for *E. coli* counts in cheeses produced from raw milk. According to a study conducted in Serbia, more than 80% of cheeses produced from raw milk contained *E. coli* counts higher than 2 log_10_ CFU/g [61]. Similarly, a study conducted in Brazil reported the presence of *E. coli* in 100% of cheeses produced from raw milk [62]. High *E. coli* counts in cheeses made from raw milk have also been reported in other studies [58,63,64].

CPS represents a frequent food safety concern in cheeses produced from raw milk [65,66,67]. In the present study, their counts were 4.69 ± 0.21 log_10_ CFU/g in *Podliveni* cheese produced from cow’s milk and slightly lower, 4.57 ± 0.16 log_10_ CFU/g, in cheese produced with the addition of donkey milk, although no statistically significant differences were observed. Faccia et al. [18] reported CPS levels ranging from 2.49 to 3.23 log_10_ CFU/g in cheeses produced from donkey milk or mixtures of donkey and goat milk. In our previous study on rolled cheese produced with donkey milk, CPS were below the limit of quantification (<1.00 log_10_ CFU/g) [16]. Our results are consistent with previously reported results for CPS counts in cheeses produced from raw cow’s milk. In a study conducted in Serbia, it was reported that in 24% of cheeses produced from raw milk, the counts of CPS were 4.02 ± 1.49 log_10_ CFU/g [61]. The potential formation of heat-resistant staphylococcal enterotoxins may occur when the number of CPS exceeds 5.0 log_10_ CFU/g, which represents a particular concern from a food safety perspective [68,69]. The criteria commonly used for evaluating the microbiological quality of cheeses produced from raw milk classify CPS counts as satisfactory when ≤4 log_10_ CFU/g, acceptable when between 4 and 5 log_10_ CFU/g, and unsatisfactory when >5 log_10_ CFU/g [61,69].

The relatively high counts of *Enterobacteriaceae*, *E. coli*, and CPS in the cheeses may be attributed to several factors, including the use of raw milk for cheese production. In addition, contamination could have occurred during production through contact with hands, equipment, or the surrounding environment where cheese processing took place. The obtained results highlight the importance of implementing enhanced hygiene measures during the production of cheeses made from raw milk. Measures necessary to reduce potential food safety risks include regular monitoring of raw milk quality, proper sanitation of equipment, careful handling during cheese production, and routine microbiological testing of the final product [50].

These results indicate that the incorporation of donkey milk did not compromise the microbiological safety of *Podliveni* cheese.

### 3.3. Chemical Composition and Mineral Profile of Podliveni Cheese

The composition and pH value of *Podliveni* cheese produced from cow’s milk and *Podliveni* cheese containing 30% donkey milk are presented in Table 3, while the concentrations of essential minerals and trace elements in these cheeses are shown in Table 4. Analysis of variance (ANOVA) indicated that there were no statistically significant differences between the cheese types for the following parameters: ash content and pH value. This conclusion is based on the fact that the calculated F-values were lower than the corresponding critical F-values and the *p*-values exceeded the threshold of 0.05 (Table 3 and Table 4). On the other hand, statistically significant differences were observed between the cheese types for dry matter content, fat content, fat in dry matter, fat-free dry matter, protein content, salt content, as well as for the concentrations of Ca, P, Na, K, Mg, Zn, Cu, Fe, and the Ca/P ratio. For these parameters, the calculated F-values were higher than the critical F-values and the associated *p*-values were below 0.05.

The addition of 30% donkey milk had a statistically significant effect on the following chemical composition parameters of *Podliveni* cheese: dry matter (*p* < 0.05), fat percentage (*p* < 0.05), fat in dry matter (*p* < 0.05), fat-free dry matter (*p* < 0.05), protein (*p* < 0.05), and salt content (*p* < 0.05), as shown in Table 3. Previous studies investigating the chemical composition of donkey milk and its differences compared to cow’s milk have demonstrated that donkey milk contains lower levels of dry matter, fat, and protein than cow’s milk [7,16,26,70]. Therefore, the observed changes in cheese composition were expected and can be primarily attributed to the dilution effect caused by the higher moisture content and lower fat and protein levels in donkey milk. In addition, differences in protein composition and casein content may influence water retention and curd structure, further affecting the final composition of the cheese.

In our previous research, the inclusion of 10% and 20% donkey milk in the production of rolled cheese resulted in a reduction in dry matter content in cheeses containing donkey milk. However, these differences were not statistically significant [16]. In the present study, the proportion of added donkey milk was higher. Since donkey milk is known to have a lower dry matter content [13,71,72,73,74] compared to cow’s milk, the addition of a higher percentage of donkey milk in the production of *Podliveni* cheese contributed to a statistically significant difference (*p* < 0.05) in dry matter content. Our previous research showed that donkey milk from the Special Nature Reserve Zasavica contained between 7.20% and 9.52% dry matter [16,26].

The fat content in *Podliveni* cheese with the addition of donkey milk was approximately 5% lower, which was statistically significant (*p* < 0.05) compared to the fat content in cheese produced exclusively from cow’s milk. In our previous study, the addition of donkey milk at levels of 10% and 20% during cheese production also led to a statistically significant reduction in fat content in the final product [16]. The fat content of donkey milk from the Special Nature Reserve Zasavica ranged from 0.10% to 1.0% in our previous investigations [16,26].

Fat content in dry matter is an important parameter, as cheese labeling and classification are regulated according to this parameter under the national regulation [75]. The fat content in dry matter differed significantly in the cheese with added donkey milk. However, according to the national regulation, based on this parameter both cheeses were classified as full-fat cheeses.

The protein content of donkey milk from the Special Nature Reserve Zasavica ranged from 1.17% to 2.07% [16,26] and is lower than the protein content of cow’s milk [5,16]. However, in the present study, the protein content was statistically significantly higher in *Podliveni* cheese with the addition of donkey milk compared to cheese produced exclusively from cow’s milk. In our previous research, we also observed that protein content increased with a higher proportion of donkey milk in rolled cheese, although those differences were not statistically significant [16]. Notably, no statistically significant differences in moisture content were observed in that study, suggesting that factors other than moisture content, such as protein retention during curd formation, may contribute to this trend.

In the current study, the incorporation of a higher percentage of donkey milk resulted in a statistically significant difference in protein content. This may be related to the differences in curd structure and protein retention during cheese production. In addition, cheese containing donkey milk showed a significantly higher dry matter content, which may have contributed to an increased protein concentration expressed on a mass basis. Furthermore, the lower casein content and specific protein composition of donkey milk may influence curd structure and whey separation, affecting protein retention in the final cheese.

The protein values obtained in the present study (Table 3) were lower than those reported in some previous studies on *Podliveni* cheese [1]. Notably, lower protein values were observed in both cheese types, including cheese produced exclusively from cow milk, indicating that this observation is not solely related to the addition of donkey milk. The protein content of the raw milk used in this study (Table 1) was within the expected physiological range, supporting the reliability of the obtained results. Although comparable dry matter values were observed, differences in protein content may be attributed to variations in cheese-making technology. In particular, differences in curd cutting size, whey drainage duration, and pressing conditions may significantly influence protein retention in the curd. In the present study, smaller curd particles and extended drainage time may have contributed to increased loss of fine protein particles into whey, resulting in lower protein content in the final cheese.

The difference in ash content was not statistically significant between the cheese produced exclusively from cow’s milk and the cheese with added donkey milk. The ash content of donkey milk ranges from 0.28% to 0.51% [13,16,70,76]) and is almost two times lower than that of cow’s milk.

Salt content showed a statistically significant difference and was higher in the *Podliveni* cheese containing donkey milk. However, it should be noted that salting was performed manually, which represents a limitation of the study and may have contributed to variability in salt distribution between samples. Therefore, the observed differences in salt content should be interpreted with caution. Similar results were obtained in our previous study [16], where salting was also carried out manually.

In previous studies, the pH value of donkey milk ranged from 6.82 to 7.46 [16,26,74,77,78]. In the present study, the pH value was slightly higher in the cheese with added donkey milk, although the difference was not statistically significant, which is consistent with our previous findings [16]. Donkey milk is generally characterized by a higher pH compared to cow’s milk, which likely contributed to the slightly higher pH observed in the cheese containing donkey milk compared to cheese produced solely from cow’s milk.

The obtained results indicate that the incorporation of donkey milk may influence the nutritional profile of *Podliveni* cheese and contribute to the development of value-added traditional dairy products.

When the results of the present study are considered together with our previous findings [16], a progressive influence of increasing donkey milk proportion (10%, 20%, and 30%) on cheese characteristics can be observed. Lower inclusion levels (10% and 20%) resulted in measurable but generally less pronounced changes in cheese composition and technological performance. In contrast, the higher inclusion level used in the present study (30%) led to statistically significant changes in several compositional parameters and more pronounced technological effects, including prolonged coagulation time and lower cheese yield. In addition, preliminary comparison with previous findings suggests that higher proportions of donkey milk may also influence sensory characteristics, particularly texture and overall acceptability, which are discussed in detail in the following section. These observations indicate a dose-dependent effect of donkey milk incorporation, highlighting the importance of optimizing the proportion of donkey milk to balance technological feasibility, nutritional modification, and sensory acceptability.

A limitation of the present study is that donkey milk samples were obtained as pooled milk from Balkan and Banat donkey breeds, which did not allow differentiation between breed-specific compositional characteristics.

The calcium content was significantly lower in *Podliveni* cheese produced with the addition of 30% donkey milk (*p* < 0.05). In our previous research, the addition of donkey milk also resulted in a significant decrease in calcium content in cheese [16]. Studies investigating donkey milk have shown that its calcium content is considerably lower than that of cow’s milk [16,27].

Regarding phosphorus, its content was significantly higher in cheese produced exclusively from cow’s milk (*p* < 0.05). In our previous study, phosphorus content was also higher in rolled cheese produced solely from cow’s milk, but the difference was not statistically significant [16]. Previous studies have shown that the phosphorus content in donkey milk ranges from 355.6 to 533.3 mg/kg [27], whereas in cow’s milk it ranges from 572.82 to 1155.34 mg/kg [79].

The Ca/P ratio was significantly higher in *Podliveni* cheese produced exclusively from cow’s milk compared to cheese containing donkey milk, which is consistent with the results obtained in our previous study [16]. This parameter is considered important due to its role in optimal mineral absorption and bioavailability [80].

Sodium content was significantly higher in cheese produced with the addition of donkey milk, most likely due to manual salting. This methodological aspect should be considered a limitation when interpreting differences in sodium content between cheese types. In contrast, potassium content was significantly higher in cheese produced exclusively from cow’s milk. Previous studies have shown that the sodium content in donkey milk ranges from 355.6 to 1730 mg/kg [16,27,79,81,82,83], whereas the sodium content in cow’s milk is approximately 563.11 mg/kg [79]. The potassium content in donkey milk ranges from 444.4 to 1102.7 mg/kg [16,27,81,82,83], while in cow’s milk it ranges from 1029.13 to 1582.52 mg/kg [79].

Magnesium content was significantly higher in cheese containing donkey milk. In our previous study, the addition of 10% and 20% donkey milk did not result in significant changes in magnesium content [16]. Analyses of donkey milk have shown that magnesium content is generally lower in donkey milk than in cow’s milk, with values of 79.4 mg/kg in donkey milk and 144 mg/kg in cow’s milk [16].

Zinc content was significantly higher in *Podliveni* cheese produced exclusively from cow’s milk, and the same trend was observed for copper content. This differs from our previous study, in which the addition of 10% and 20% donkey milk during rolled cheese production did not significantly affect the concentrations of these elements [16]. In that study, zinc content in cow’s milk was 3.01 mg/kg, compared to 2.19 mg/kg in donkey milk, while copper content was 0.43 mg/kg in cow’s milk and 0.42 mg/kg in donkey milk.

In the present study, iron content was significantly higher in *Podliveni* cheese containing donkey milk. The same result was observed in our previous study, where increasing the proportion of donkey milk (10% and 20%) led to higher iron content in rolled cheese [16]. In that study, the iron content in cow’s milk was 0.37 mg/kg, whereas donkey milk contained 0.22 mg/kg.

### 3.4. Sensory Evaluation of Podliveni Cheese

The sensory parameters and panelists’ scores for *Podliveni* cheeses are presented in Table 5.

A statistically significant difference was observed for texture between the two cheeses (*p* = 0.040), with higher scores recorded for the cheese produced exclusively from cow’s milk. The lower sensory scores may be related to the softer texture and higher whey separation observed in cheeses containing donkey milk, which may be related to the lower casein content and different protein composition of donkey milk affecting curd formation and the structural properties of cheese [11,38,39]. No statistically significant differences were detected for color, aroma, taste, or overall liking (*p* > 0.05). However, for all evaluated sensory parameters (color, aroma, taste, and overall liking), slightly higher scores were generally recorded for *Podliveni* cheese produced solely from cow’s milk. The lower sensory scores for *Podliveni* cheese produced with the addition of donkey milk may also be associated with its lower fat content, which, in combination with higher moisture content, may negatively affect the overall sensory perception of the product [84]. Although flavor was not assessed through a dedicated hedonic flavor scale, the combination of aroma and taste scores, together with participants’ qualitative feedback, suggested subtle sensory differences between the samples. *Podliveni* cheese containing 30% donkey milk was perceived as slightly more acidic and mildly sweeter, accompanied by softer chewiness and a more delicate structure compared to *Podliveni* cheese produced exclusively from cow milk.

Overall, the sensory scores obtained in this study were somewhat lower compared to those reported in our previous research on the acceptability of rolled cheese [16]. In that study, the addition of donkey milk at levels of 10% and 20% resulted in slightly higher sensory scores compared to rolled cheese produced exclusively from cow’s milk, although the differences were not statistically significant. Similarly, studies conducted by other authors have shown that the addition of donkey milk in smaller proportions (5–10%) may improve the sensory acceptability of cheeses to which it is added [22,85]. The generally lower sensory scores observed in the present study may be related to the softer texture, higher moisture content, and increased whey separation observed in cheeses containing donkey milk. These characteristics may influence the overall perception of the product. The lower sensory scores may also be related to the relatively high *Enterobacteriaceae* counts observed in the present study, as higher levels of these bacteria are known to negatively affect the sensory properties of cheese [86,87]. Nevertheless, the absence of statistically significant differences for most sensory parameters indicates that the addition of donkey milk did not significantly reduce sensory acceptability.

A limitation of this study is that each experimental group was represented by a single cheese sample due to the limited availability of donkey milk produced under extensive farming conditions. Nevertheless, all analytical measurements were performed in triplicate to ensure analytical precision and reliability of the obtained results. Therefore, the statistical analysis primarily reflects analytical variability rather than biological replication. Future studies including multiple independent production batches are recommended to further validate these findings.

## 4. Conclusions

The obtained results demonstrated that the incorporation of donkey milk from the autochthonous Balkan and Banat donkey breeds into the production of traditional *Podliveni* cheese is feasible and does not result in major changes in microbiological quality or in most sensory characteristics. However, the observed microbiological counts highlight the importance of strict hygiene control during production, particularly when raw milk is used.

The addition of donkey milk at a level of 30% significantly affected coagulation time, as well as the chemical and mineral composition of the cheese. Future studies should focus on technological optimization strategies to improve curd formation, yield, and texture of cheeses containing higher proportions of donkey milk. Potential approaches may include optimization of coagulation temperature and time, optimization of rennet dosage, the use of appropriate starter cultures, and evaluation of different inclusion levels and processing modifications to define optimal production conditions. Nevertheless, the results obtained in this study provide a valuable basis for further improvement and development of this and other dairy products containing donkey milk and may contribute to the valorization and sustainable use of autochthonous donkey breeds.

## Figures and Tables

**Table 1:** Physicochemical and microbiological characteristics of donkey’s and cow’s milk used for cheese production (means ± S.D.) (adapted from [16]).

Parameter (Unit)	Donkey’s Milk	Cow’s Milk
Fat (%)	0.45 ± 0.29	3.93 ± 0.32
Protein (%)	1.67 ± 0.20	3.42 ± 0.07
Moisture (%)	91.4 ± 0.42	87.2 ± 0.15
Ash (%)	0.38 ± 0.05	0.61 ± 0.01
pH	7.23 ± 0.14	6.64 ± 0.02
Total mesophilic bacteria (log10 CFU/g)	5.10 ± 1.57	4.31 ± 0.20

**Table 2:** Microbiological status of *Podliveni* cheeses (Means ± S.D.).

Parameter	*Podliveni* Cheese with Cow’s Milk	*Podliveni* Cheese with the Addition of 30% Donkey’s Milk	ANOVA	
			*p*-Value	F
Lactic acid bacteria (log_10_ CFU/g)	8.83 ± 0.32	8.93 ± 0.05	0.433	0.669
*Enterobacteriaceae* (log_10_ CFU/g)	4.59 ± 0.25	4.94 ± 0.32	0.059	4.519
*Escherichia coli* (log_10_ CFU/g)	3.03 ± 0.23	2.98 ± 0.124	0.612	0.275
Coagulase-positive staphylococci (log_10_ CFU/g)	4.69 ± 0.21	4.57 ± 0.16	0.289	1.270
*Salmonella* spp. (25 g)	Not detected	Not detected	-	-
*Listeria monocytogenes* (25 g)	Not detected	Not detected	-	-

**Table 3:** Composition and pH of cheeses (Means ± S.D.).

Composition (Unit)	*Podliveni* Cheese with Cow’s Milk	*Podliveni* Cheese with the Addition of 30% Donkey’s Milk	ANOVA	
			*p*-Value	F
Dry matter (%)	39.82 ± 0.26	40.72 ± 0.25	0.012	19.1
Fat (%)	22.00 ± 0.50	20.87 ± 0.32	0.030	10.9
Fat in dry matter (%)	55.24 ± 1.15	51.25 ± 0.60	0.006	28.4
Fat-free dry matter (%)	77.13 ± 0.42	74.91 ± 0.21	0.001	66.6
Protein (%)	13.45 ± 0.09	15.75 ± 0.05	<0.001	1511
Ash (%)	1.54 ± 0.04	1.52 ± 0.06	0.712	0.16
pH	4.86 ± 0.06	4.94 ± 0.06	0.165	2.87
Salt (%)	0.25 ± 0.01	0.27 ± 0.01	0.025	12.3

**Table 4:** Essential minerals and trace elements (mg/kg) in cheeses (Means ± S.D.).

Elements (Unit)	*Podliveni* Cheese with Cow’s Milk	*Podliveni* Cheese with the Addition of 30% Donkey’s Milk	ANOVA	
			*p*-Value	F
Ca ^1^ (mg/kg)	3577 ± 26.2	3276 ± 25.9	<0.001	200
P ^2^ (mg/kg)	2860 ± 22.7	2791 ± 26.3	0.026	11.9
Na ^3^ (mg/kg)	998 ± 7.00	1094 ± 23.7	0.003	45.5
K ^4^ (mg/kg)	694 ± 11.4	506 ± 7.02	<0.001	592
Mg ^5^ (mg/kg)	101 ± 5.86	127 ± 4.58	0.004	37.6
Zn ^6^ (mg/kg)	24.9 ± 0.90	19.5 ± 0.84	0.002	58.5
Cu ^7^ (mg/kg)	8.68 ± 0.17	7.85 ± 0.29	0.013	18.5
Fe ^8^ (mg/kg)	5.75 ± 0.12	6.74 ± 0.10	<0.001	121
Ca/P ratio	1.25 ± 0.02	1.17 ± 0.01	0.005	31.8

Notes: ^1^ Ca—calcium; ^2^ P—phosphorus; ^3^ Na—sodium; ^4^ K—potassium; ^5^ Mg—magnesium; ^6^ Zn—zinc; ^7^ Cu—copper; ^8^ Fe—iron. The results are presented as the mean value and standard deviation (SD) (*n* = 3); *F* crit = 7.71.

**Table 5:** Consumer acceptability of *Podliveni* cheeses (Means ± S.D.).

Parameter	*Podliveni* Cheese with Cow’s Milk	*Podliveni* Cheese with the Addition of 30% Donkey’s Milk	ANOVA	
			*p*-Value	F
Color	3.96 ± 1.17	3.90 ± 1.19	0.899	0.016
Texture	3.38 ± 1.21	2.67 ± 0.89	0.040	4.5
Aroma	3.43 ± 1.18	3.10 ± 1.11	0.362	0.849
Taste	3.24 ± 1.23	2.86 ± 1.21	0.329	0.977
Overall liking	3.19 ± 1.22	2.95± 1.00	0.503	0.456

## Data Availability

The original contributions presented in the study are included in the article; further inquiries can be directed to the corresponding authors.

## Abbreviations

The following abbreviations are used in this manuscript:

LAB	Lactic acid bacteria
CPS	Coagulase-positive staphylococci
